# Local Control by Radiofrequency Thermal Ablation Increased Overall Survival in Patients With Refractory Liver Metastases of Colorectal Cancer

**DOI:** 10.1097/MD.0000000000003338

**Published:** 2016-04-08

**Authors:** Po-Chih Yang, Been-Ren Lin, Yi-Chang Chen, Yu-Lin Lin, Hong-Shiee Lai, Kai-Wen Huang, Jin-Tung Liang

**Affiliations:** From the Department of Surgery, National Taiwan University Hospital Hsinchu Branch, Hsinchu (P-CY); Department of Surgery (B-RL, H-SL, K-WH, J-TL); Department of Medical Imaging (Y-CC); and Department of Oncology, National Taiwan University Hospital and College of Medicine, National Taiwan University, Taipei, Taiwan, ROC (Y-LL).

## Abstract

Radiofrequency thermal ablation (RFA) is widely used for local solitary liver tumor control. However, the benefit of RFA for colorectal cancer with liver metastases, which is refractory to chemotherapy, remains unknown.

We retrospectively enrolled 70 consecutive colorectal adenocarcinoma patients, who had synchronous liver metastases, who were refractory to chemotherapy, and whose life expectancy was >6 months, into this study to investigate the outcomes of RFA and associated prognostic factors. RFA was introduced to all of these patients during the enrollment. The time interval from RFA to recurrence of liver metastases and overall survival was recorded. Age, sex, carcinoembryonic antigen level, primary tumor location, postoperative adjuvant chemotherapy regimens, and the size and number of metastatic liver lesions were recorded. Cox regression analysis was used to determine the prognostic significance.

Thirty-nine patients accepted RFA during chemotherapy, whereas 31 chose to receive chemotherapy alone. Patients with ≤5 and >5 liver metastases had median survival durations of 28 and 17 months, respectively (*P* = 0.018). The dominant liver tumor size (<5 vs ≥5 cm) was significantly associated with median survival (30 vs 17 months, respectively; *P* = 0.038), as was the carcinoembryonic antigen level (35 vs 16 months for ≤200 vs >200 ng/mL respectively; *P* = 0.029). Besides, radiofrequency thermal ablation plus chemotherapy was associated with a better median overall survival than chemotherapy alone (29 vs 12 months, respectively; *P* = 0.002). In multivariate analysis, only radiofrequency thermal ablation treatment and number of liver tumors were significant prognostic factors for survival. Our result further revealed that patients treated with radiofrequency thermal ablation had longer progression-free intervals than those treated with chemotherapy alone (18 vs 9 months, respectively; *P* = 0.001). Hence, radiofrequency thermal ablation is a safe and effective adjunct treatment to chemotherapy.

## INTRODUCTION

Colorectal cancer (CRC) is a devastating disease. Based on the statistical data, there were about 25% of CRC patients having synchronous liver metastases while being diagnosed, whereas another 50% eventually developed recurrent disease within their livers.^1,2^ Complete resection for liver metastases remains the criterion standard of treatment for CRC with liver metastases. However, 80% to 90% of them, unfortunately, are usually ineligible to receive complete resection because of either their extensive liver lesions or multiple medical comorbidities.^3,4^ Palliative chemotherapy is the standard of care for metastatic CRC patients who are ineligible to receive complete resection of liver metastases in the past.^5^ Nevertheless, there were growing evidence showing that regional treatments, including intrahepatic arterial infusion pumps, cryotherapy, chemoembolization, and radiofrequency thermal ablation (RFA), may provide some benefits to those patients who have inoperable liver metastases.

RFA is widely used for local control of primary and secondary liver tumors. During RFA, heat, generated from a high-frequency alternating current, is applied to induce cellular death. Several studies^6–8^ showed that RFA is safe and feasible in patients with solitary metastatic liver tumors. They had further shown that overall survival (OS) rates were not significantly different between patients who underwent RFA and those who underwent surgical resection of liver metastases.^6–8^ In terms of cases involving multiple liver lesions that would like to treat with curative intent, however, it remains unclear whether surgery to liver metastases or RFA may provide better OS. Some studies^9–13^ reported that RFA-treated patients have similar OS when compared with surgically treated patients, whereas other studies showed better OS rates in surgically treated patients.

Although benefits of RFA for patients who had resectable solitary liver metastases have been proven, the outcomes of RFA to those patients who had unresectable CRC with liver metastases and only received palliative chemotherapy remain unknown. Therefore, the aim of this study was to evaluate the potential benefits of RFA plus chemotherapy compared with that of chemotherapy alone for CRC with metastatic lesions confined to the liver. Additionally, we evaluated various factors that may predict survival in these patients.

## MATERIAL AND METHODS

We retrospectively enrolled consecutive patients, whose cancers were histologically proven as adenocarcinoma of the colon or rectum, who had synchronous liver metastases, and were referred to the National Taiwan University Hospital between January 2007 and December 2009. Their treatments were evaluated and decided by a multidisciplinary team, which included colorectal surgeons, liver surgeons, oncologists, radiologists, and pathologists. The reasons for not being able to perform complete hepatic resection of liver metastases included the number and location of liver lesions, insufficient hepatic reserve of patients, and patients’ comorbidities. Patients were considered to be potential candidates for RFA treatment if they had the following 2 conditions and responded poorly to chemotherapy (determined by growing or new liver masses identified by computed tomography [CT] or by elevated serum carcinoembryonic antigen [CEA] levels): they still had liver metastases after their primary cancers had been resected and they had received at least 2 different regimens of chemotherapy (generally consisting of FOLFOX or FOLFIRI ± bevacizumab), and they remained in good performance status (Eastern Cooperative Oncology Group-World Health Organization scores of 0 or 1). Patients who had either a life expectancy of <6 months or had their disease progressed outside their livers were excluded. All of the patients who agreed to receive RFA continued to receive chemotherapy during and after RFA. The regimens during RFA treatment between these 2 groups are FOLFOXIRI or high-dose infusional 5 FU/leucovorin ± targeted therapy.

RFA was performed percutaneously for hepatic tumors <5 cm under ultrasonographic guidance. All of the patients received RFA with general anesthesia in the operation room. Single 17-G internally cooled electrodes (Cool-tip^TM^ RF ablation system, COVIDIEN, Mansfield, Massachusetts, USA) were used for each tumors. The radiofrequency current had been applied for 12 minutes for each tumor. Either 1 or 2 tumor was ablated in each RFA sessions depending on the total number of the tumors. Follow-up CT scanning of the ablated tumors was proceeded 1 month after RFA. Patients underwent additional RFA sessions for those who had the residual tumors, which was unable to be ablated completely in the first attempt, or for those whose viable tumors after ablation remained identified based on CT scan. All the patients who only received chemotherapy were followed-up by abdominal CT scanning every 3 months. The numbers and sizes of metastatic hepatic tumors were reviewed via CT by a single radiologist. The response to treatment was scored retrospectively as partial response, stable disease, or progressive disease (PD) according to the revised response evaluation criteria in solid tumors (RECIST) guideline.^14^ Recurrence from previously ablated tumor was evaluated and reablated by the same surgeon.

A number of potential prognostic variables were analyzed. All patient data were obtained and managed in accordance with the approved guidelines from the Institutional Review Board of the National Taiwan University Hospital. The progression-free time from RFA to recurrence of liver metastases was determined from RFA application to PD. The time of progression-free from RFA to recurrence of liver metastases and OS were analyzed by the Kaplan-Meier curve. The difference between RFA plus chemotherapy and chemotherapy alone was evaluated by the log-rank test. The associations among prognostic variables with progression-free time from RFA to recurrence of liver metastases and OS were analyzed by the Cox proportional-hazards model. Data were analyzed using SPSS 19.0 statistical software (SPSS Inc, Chicago, IL). *P* values <0.05 were considered statistically significant.

## RESULTS

### Demographics

Thirty-nine patients were enrolled into the RFA plus chemotherapy group and 31 were enrolled in the chemotherapy alone group. Patients’ characteristics were presented in Table 1. The 2 groups had similar mean ages, serum CEA levels, distributions of primary and metastatic tumor locations, frequencies of comorbid medical conditions, numbers of liver metastases, largest size of hepatic metastases, and frequencies of receiving targeted therapy.

**TABLE 1:**
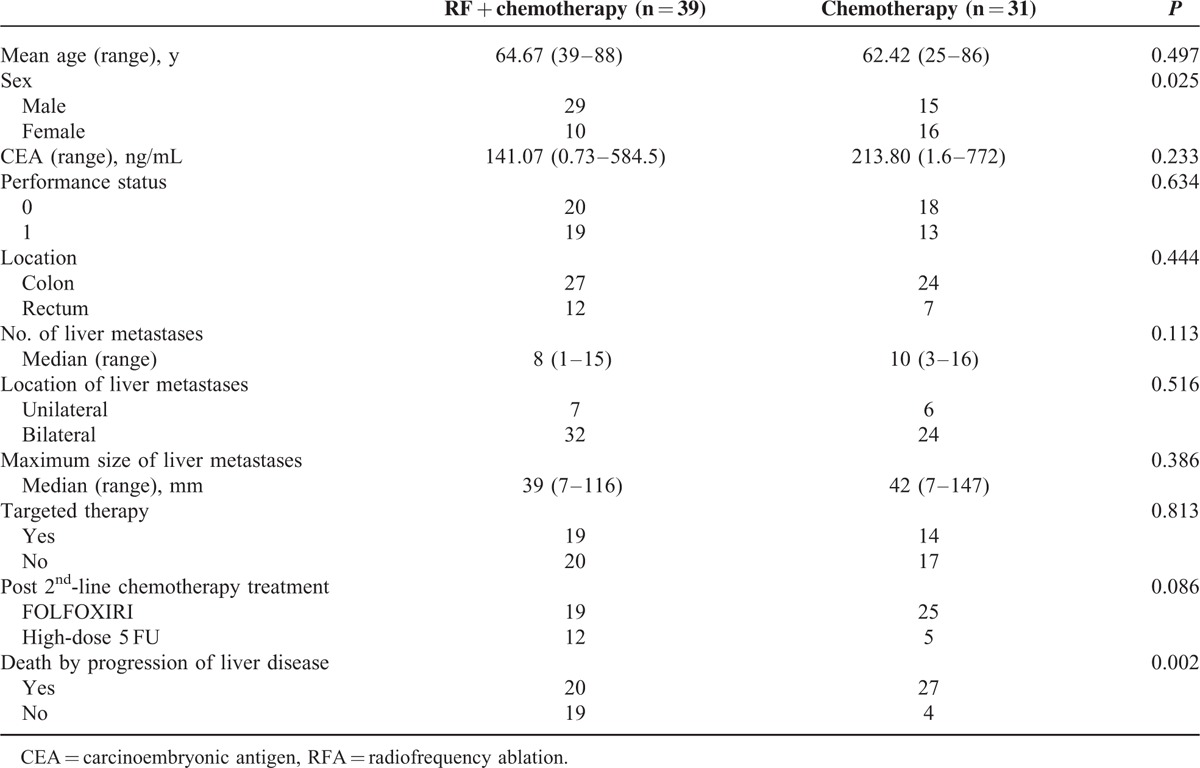
Distribution and Clinical Characteristics of Patients in the RFA Plus Chemotherapy and Chemotherapy-alone Treatment Groups

The surgical intervention for primary colon cancer was also analyzed. In RFA plus chemotherapy group, among 27 patients with primary cancer in colon, 5 received anterior resection, 10 received left hemicolectomy, and 12 received right hemicolectomy. While other 12 patients with primary rectal cancer, all of them received low anterior resection. In chemotherapy-alone group, 7 received anterior resection for their sigmoid colon cancers, 7 received left hemicolectomy, 10 received right hemicolectomy, and 7 received low anterior resection for their rectal cancers. Eighty-five percent (33/39) of operations in RFA plus chemotherapy group were conducted by laparoscopic method, whereas 87% (27/31) of operations in chemotherapy group were performed by the same method (laparoscopy) (*P* = 0.77).

### RFA Result

A total of 113 RFA sessions for 135 tumors were performed in 39 patients. The mean number of RFA sessions for each patient was 2.89 (range: 1–11, SD: 2.26). The mean size of an ablated tumor was 2.96 cm (range: 0.7–4.8 cm, SD: 1.12). The complete ablation rate was 91.9% (124/135). Seven patients received additional RFA because of 9 recurrent tumors. The median recurrent time of these 9 tumors is 6 months. While most patients received RFA for >1 time because multiple tumors, which were unable to ablate in the first attempt, but not because of recurrent tumors. Post-RFA complications were assessed and graded according to the previously described classification of surgical complications.^15^ The complication rate was 6.19% (7/113), with complications including the formation of 3 hepatic abscesses without drainage (Grade I), 2 pleural effusions (Grade IIIa), 1 hemoperitoneum (Grade II), and 1 hepatic abscess with radiological drainage (Grade IIIa). There were no cases of post-RFA mortality during the same admission period. The patients in chemotherapy-alone group continued to receive different cycles of chemotherapy and their common morbidities were neutropenia, anemia, and bacteremia. The mortality in these patients was caused by progression of liver disease.

### Overall and Progression-Free Survival

**FIGURE 1:**
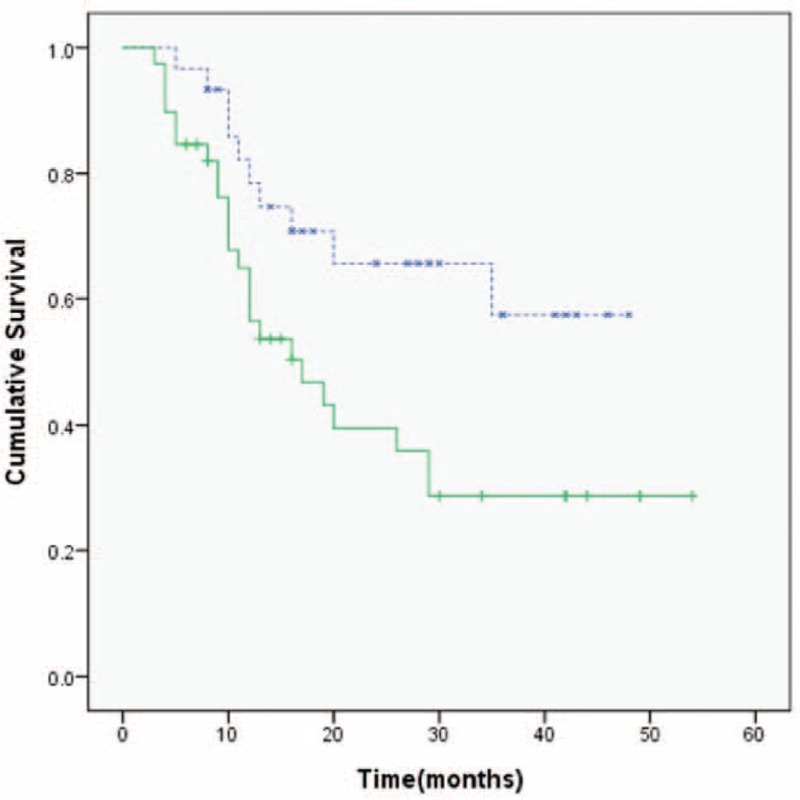
Kaplan-Meier survival analysis according to the number of liver metastases. Patients with ≤5 metastatic liver tumors (blue lines) had a longer survival duration than those with >5 liver tumors (green lines) (median survival: 28 vs 17 months, respectively; *P* = 0.018).

**FIGURE 2:**
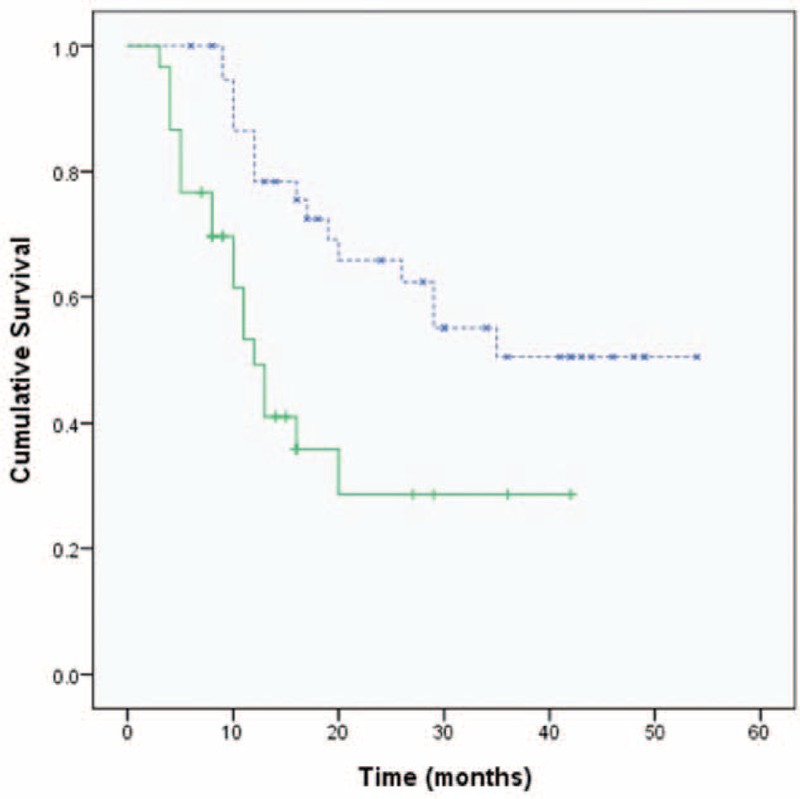
Kaplan-Meier plot showing overall survival analysis according to treatment method. The radiofrequency ablation (RFA) plus chemotherapy group (blue lines) has longer overall survival than the chemotherapy-alone group (green lines) (median survival: 29 vs 12 months, respectively; *P* = 0.002).

Patients with <5 liver metastatic tumors had a longer duration of survival than those with >5 liver tumors (28 vs 17 months, respectively; *P* = 0.018; Figure 1). Patients with dominant lesions <5 cm in size had better survival than those with dominant lesions >5 cm (30 vs 17 months respectively; *P* = 0.038). Serum CEA levels less than 200 ng/mL were also associated with better survival than those >200 ng/mL (35 vs 16 months, respectively; *P* = 0.029). Overall survival in the group treated with RFA plus chemotherapy was significantly longer than that in the chemotherapy-alone group (29 vs 12 months, respectively, *P* = 0.002; Figure 2). No survival advantage was observed with respect to sex, age, medical comorbidity, colon versus rectal primary tumor location, or the use of targeted therapy, as shown in Table 2. In multivariate analysis, the independent factors associated with survival were limited to treatment type and the number of liver tumors. Only RFA treatment and metastatic liver tumors ≤5 were significant predictors of mortality (odds ratio [OR]: 4.122, 95% confidence interval [CI]: 1.897–8.953, *P* = 0.001 and OR: 3.359, 95% CI: 1.485–7.598, *P* = 0.004; respectively), shown in Table 3. The rate of lost follow-up is 5.13% (2/39) in RFA group and 3.22% (1/31) in chemotherapy-alone group.

**TABLE 2:**
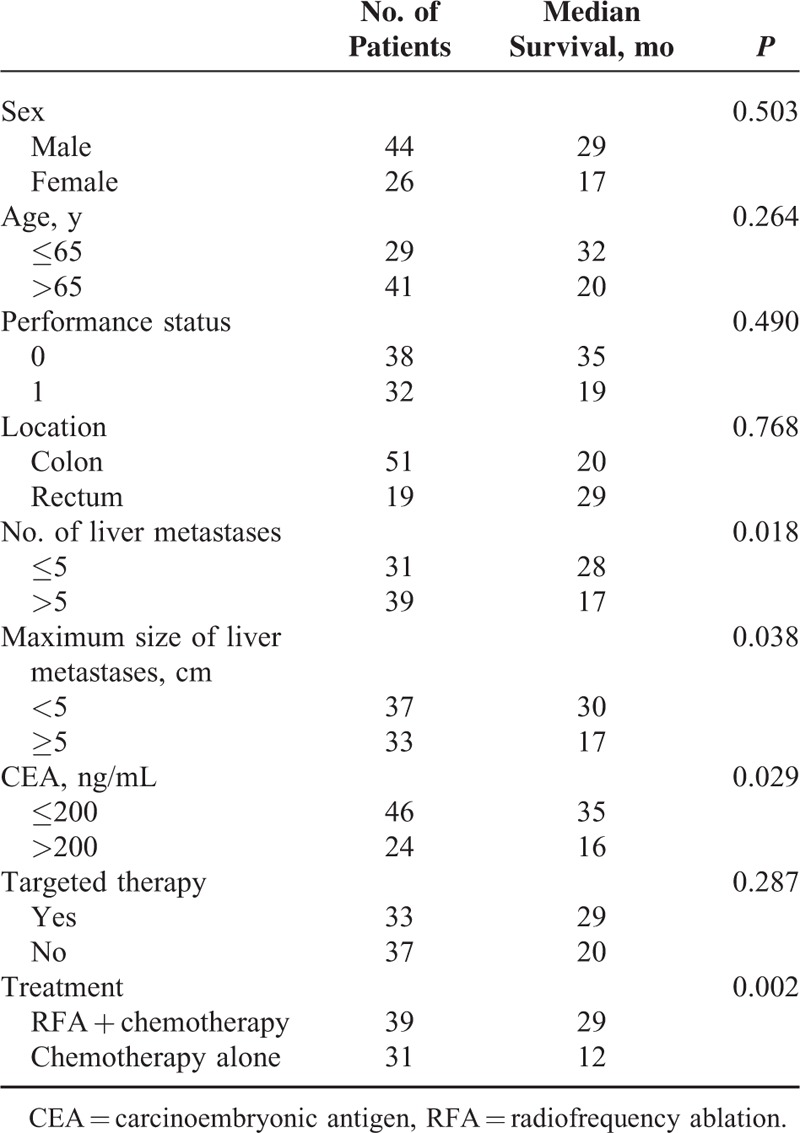
Univariate Analysis of Prognostic Factors for Overall Survival, Evaluated in all Patients (n = 70)

**TABLE 3:**
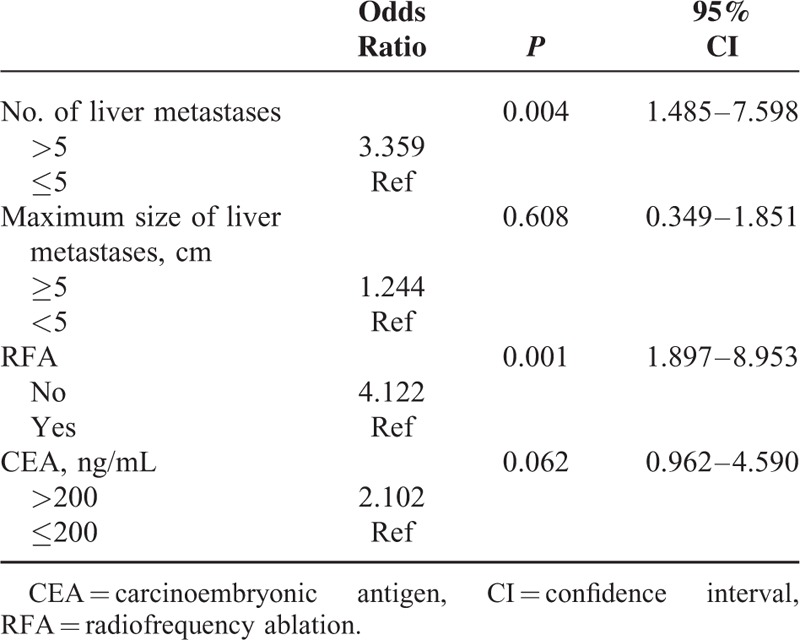
Multivariate Analysis of Overall Survival According to a Cox Proportional Hazards model

**FIGURE 3:**
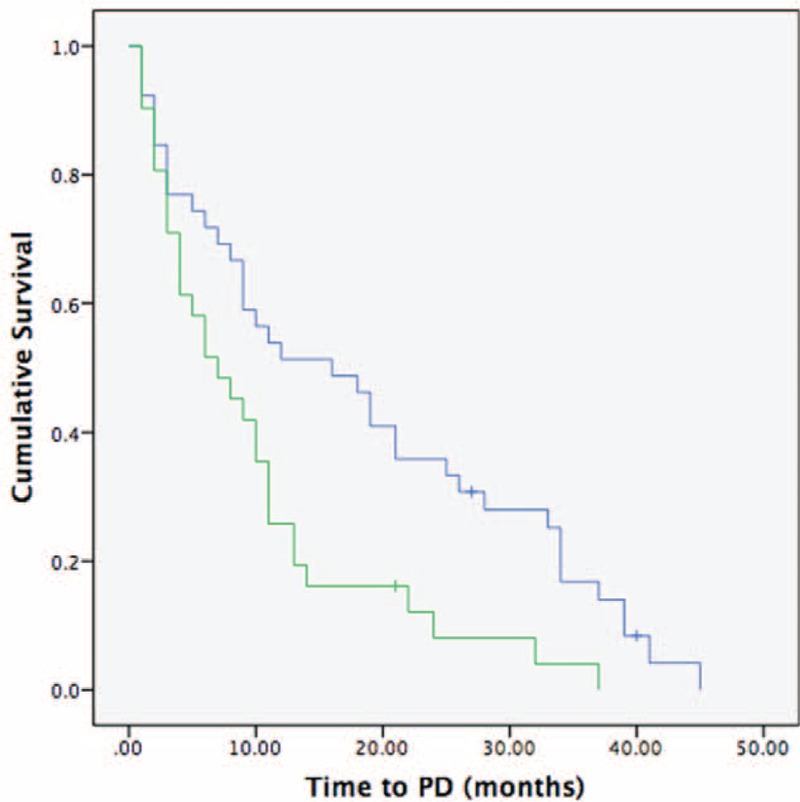
Kaplan-Meier survival analysis for progression-free survival according to treatment method. The chemotherapy-only group (green lines) has a shorter interval to progressive disease than the radiofrequency ablation (RFA) plus chemotherapy (blue lines) group (median time to progression: 9 vs 18 months, respectively; *P* = 0.001).

Our result further revealed that patients treated with RFA had longer progression-free intervals than those treated with chemotherapy alone (18 vs 9 months, respectively; *P* = 0.001), shown in Figure 3. Although most patients were died of progression of liver disease, however, RFA treatment significantly decreased the incidence of death caused by hepatic failure or biliary infection (51% in the RFA plus chemotherapy group and 87% in the chemotherapy-alone group, *P* = 0.002), shown in Table 1.

## DISCUSSION

The previous studies showed that patients with “solitary resectable” liver tumor treated with RFA has equivalent OS compared to those treated with surgical resection. RFA was used for curative intent in these studies.^6–8^ However, the outcomes of RFA to those patients who had unresectable CRC with liver metastases and only received palliative chemotherapy remain unknown.^11,12^ We are uncertain whether these patients, who are not eligible for surgical resection with curative intent, can benefit from RFA. In our study, comparison was conducted between the patients who received RFA plus chemotherapy to those who received chemotherapy alone. Therefore, we limited our study to patients with unresectable CRC with liver metastases who responded poorly to chemotherapy (determined by growing or new liver masses identified by computed tomography or by elevated serum CEA levels) and RFA was included with palliative intent. All patients continued to receive chemotherapy and RFA was added as an adjuvant therapy during the period between different cycles of chemotherapy. The regimens between these 2 groups are FOLFOXIRI or high-dose infusional 5FU/leucovorin. Notably, our data showed that more patients in the chemotherapy-alone group received FOLFOXIRI (22/30, 73.3%), which is a more intensive regimen, when they compared to those, who received RFA plus chemotherapy (19/31, 61.3%). This result indirectly demonstrated that the shorter overall survival in the chemotherapy-alone group was not attributed to a weaker intensity of chemotherapy, although the percentage of the patients who received FOLFOXIRI between 2 groups was not significantly different (*P* = 0.086). This result was shown in Table 1

Furthermore, we analyzed the cause of death in both groups. Most patients with CRC with liver metastases died from hepatic tumors progression, including hepatic failure and severe bile tract infection. Other causes of death included sepsis, multiple organ failure, or other metastases. However, the proportion of patients dying from hepatic progression was greater in the chemotherapy group than in the RFA group (87% vs 51% respectively, *P* = 0.002). This could be explained by the reasons that RFA may provide better local (hepatic) control for metastatic disease^16^ and may reduce the possibility of hepatic tumor progression. Upon analyzing 292 RFA sessions performed between 1997 and 2006, Siperstein et al^17^ also had similar results and suggested that a greater percentage of patients treated with RFA die of causes other than liver failure.

Prognostic factors associated with increased survival were identified in our study. Consistent with previous studies,^17–19^ we found that parameters associated with tumor burden, such as the CEA level, tumor size, and number of hepatic lesions, determined overall survival. Multivariate analysis also indicated that fewer hepatic lesions were significant predictors of longer survival.

Complications after RFA had been analyzed in several large case series. The frequency of major RFA complications ranges from 0.6% to 8.9%.^20,21^ In our study, the overall complication rate was 6.19%, in line with previous studies. In recent years, new devices capable of ablating tumors >5 cm have appeared; this would make more tumors in studies such as ours eligible for ablation.

We retrospectively evaluated the benefits of RFA in patients who had CRC with liver metastases and refractory to second-line chemotherapy. Therefore, this nonrandomized study, indeed, had some limitations. The limitations included the retrospective nature of data acquisition, heterogeneous patient populations, different chemotherapy regimens before RFA, and a small sample size. It could be argued that comparing the outcomes of these patients, who had received and not received RFA, is inconclusive because the disease nature of those patients who received RFA may be more indolent than those who were unable to receive RFA. This bias could also explain the relatively shorter overall and progression-free survival in the group of chemotherapy alone. However, in our study, this bias was, in part at least, overcome by subsequent comparison of the characteristics of the 2 groups shown in Table 1. Although our study identified the palliative role of RFA in patients with CRC with liver metastases, further prospective studies are required to verify the results reported here.

## CONCLUSION

The results of our study provide evidence that RFA plus chemotherapy may delay the progression of hepatic tumors and improve the survival of colorectal cancer patients when surgical resection is not feasible and metastases are confined to the liver. There was a low frequency of RFA complications and no deaths immediately following treatment. For patients with poor responses to chemotherapy, RFA could be an adjunct treatment of choice.
